# The Impact of Feedback on the Different Time Courses of Multisensory Temporal Recalibration

**DOI:** 10.1155/2017/3478742

**Published:** 2017-02-21

**Authors:** Matthew A. De Niear, Jean-Paul Noel, Mark T. Wallace

**Affiliations:** ^1^Medical Scientist Training Program, Vanderbilt University Medical School, Vanderbilt University, Nashville, TN 37235, USA; ^2^Vanderbilt Brain Institute, Vanderbilt University Medical School, Vanderbilt University, Nashville, TN 37235, USA; ^3^Neuroscience Graduate Program, Vanderbilt Brain Institute, Vanderbilt University Medical School, Vanderbilt University, Nashville, TN 37235, USA; ^4^Department of Hearing and Speech Sciences, Vanderbilt University Medical Center, Nashville, TN 37235, USA; ^5^Department of Psychology, Vanderbilt University, Nashville, TN 37235, USA; ^6^Department of Psychiatry, Vanderbilt University Medical Center, Nashville, TN 37235, USA

## Abstract

The capacity to rapidly adjust perceptual representations confers a fundamental advantage when confronted with a constantly changing world. Unexplored is how feedback regarding sensory judgments (top-down factors) interacts with sensory statistics (bottom-up factors) to drive long- and short-term recalibration of multisensory perceptual representations. Here, we examined the time course of both cumulative and rapid temporal perceptual recalibration for individuals completing an audiovisual simultaneity judgment task in which they were provided with varying degrees of feedback. We find that in the presence of feedback (as opposed to simple sensory exposure) temporal recalibration is more robust. Additionally, differential time courses are seen for cumulative and rapid recalibration dependent upon the nature of the feedback provided. Whereas cumulative recalibration effects relied more heavily on feedback that informs (i.e., negative feedback) rather than confirms (i.e., positive feedback) the judgment, rapid recalibration shows the opposite tendency. Furthermore, differential effects on rapid and cumulative recalibration were seen when the reliability of feedback was altered. Collectively, our findings illustrate that feedback signals promote and sustain audiovisual recalibration over the course of cumulative learning and enhance rapid trial-to-trial learning. Furthermore, given the differential effects seen for cumulative and rapid recalibration, these processes may function via distinct mechanisms.

## 1. Introduction

In order to accurately perceive the world, individuals must adjust their perceptual representations to meet the changing nature of the sensory world and changing task contingencies [1]. Given this, the capacity to rapidly adjust perceptual representations confers a fundamental advantage [2, 3]. Such perceptual plasticity often leads to an improved representation of the sensory environment, a process termed perceptual learning [4, 5]. Changes in perceptual representations resulting from perceptual learning have been observed to occur within both rapid [6, 7] and more gradual time courses [8, 9]. Furthermore, the contribution of feedback signals, in conjunction with sensory experience, is known to alter the rate of perceptual learning or enable perceptual learning to occur when sensory experience is insufficient [10].

Although initial investigations of perceptual plasticity tended to focus on changes in perception for a single sensory modality, there has been an increasing interest in examining the plasticity of multisensory perceptual representations [4, 11]. One such area of investigation has focused on how the temporal processing of multisensory stimuli (particularly audiovisual stimuli [12–15]; for other modalities see [16–18]) can be altered via changes in sensory experience. The temporal structure of sensory stimuli from the different modalities is a fundamental feature determining whether these stimuli should be associated or perceived as a single multisensory event [19–21]. One critical aspect of this process must take into account the differences in neural and physical transmission times for the respective sensory stimuli (e.g., light and sound energy propagate through the environment at very different rates). In order to circumvent this challenge and ultimately achieve perceptual coherence, there exists an epoch of time spanning several hundred milliseconds within which stimuli from vision and audition are likely to be associated. This construct has been collectively referred to as the temporal binding window (TBW). Similarly, the point of asynchrony at which the separate sensory stimuli are most likely to be perceived as occurring synchronously has been termed the point of subjective simultaneity (PSS). These two metrics, the TBW and PSS, are thus important tools in evaluating the nature of audiovisual temporal representations.

Prior work has shown both the TBW and PSS to be malleable. These dynamic changes in the TBW and PSS, termed temporal recalibration, were initially hypothesized as a means to resolve asynchronous sensory signals reflective of the statistics of the environment [19]. Thus, initial studies showed that it was possible to shift an individual's PSS by providing extensive experience that overrepresented certain asynchronies [12, 13]. More recent evidence suggests that these changes not only occur after extensive experience, but can also be seen on a moment-to-moment basis (i.e., based on the characteristics of the previous trial, t-1) [7, 15, 22–27]. Thus, changes in multisensory temporal representations happen on both rapid and cumulative time scales. Such observations raise fundamental mechanistic questions about these short- and longer-term changes, most immediately in regard to whether one (short-term) represents the substrate upon which the other (longer-term) is built. One can envision a scenario in which rapid temporal recalibration may be needed in order to properly represent immediate changes in the sensory environment whereas cumulative temporal recalibration may result in more durable changes in perceptual representations [24, 28].

While sensory experience is undoubtedly an important element that influences perceptual plasticity, feedback signals that inform an individual regarding the accuracy of their perceptual judgments are likely to interact with sensory experience to influence temporal recalibration of the TBW and PSS. Early studies of visual perceptual learning suggest that feedback signals enhance perceptual learning [9, 29] and are capable of eliciting perceptual learning even in the absence of awareness in regard to the changing nature of the sensory environment [30]. Increasingly, changes on top-down processing regions have been observed to parallel perceptual learning [31, 32] and are likely to be activated by a feedback signal. Collectively, the evidence suggests that the dynamics of perceptual learning are likely dependent upon coordinated interactions between sensory statistics primarily represented in low-level cortical areas and the brain areas that initially represent them and higher-order factors and their neural substrates [33]. Recent studies have observed that feedback signals also produce rapid improvements in multisensory temporal acuity [34–36] and elicit changes in connectivity between primary sensory cortices and multisensory cortex [37]. Despite the independent evidence for the importance of these bottom-up and top-down factors in perceptual plasticity, few studies have looked at the interdependence between them. Here, we sought the interaction of bottom-up and top-down factors in perceptual plasticity by altering top-down factors (i.e., presence of a feedback signal and/or feedback reliability) and examining its impact on temporal recalibration across both immediate and longer-term time scales.

## 2. Materials and Methods

### 2.1. Participants

Sixty-five young adults partook in this study (36 females; age, *M* = 20.48 years; range = 18–28 years). All participants had self-reported normal hearing and normal or corrected to normal vision. Written informed consent was obtained from all individuals participating in this study. All participant recruitment and experimental procedures were approved by the Vanderbilt University Institutional Review Board and were in accordance with the ethical standards of the 1964 Helsinki Declaration and its later amendments or comparable ethical standards.

### 2.2. Assessment of Temporal Acuity by Simultaneity Judgment Task

**Figure 1:**
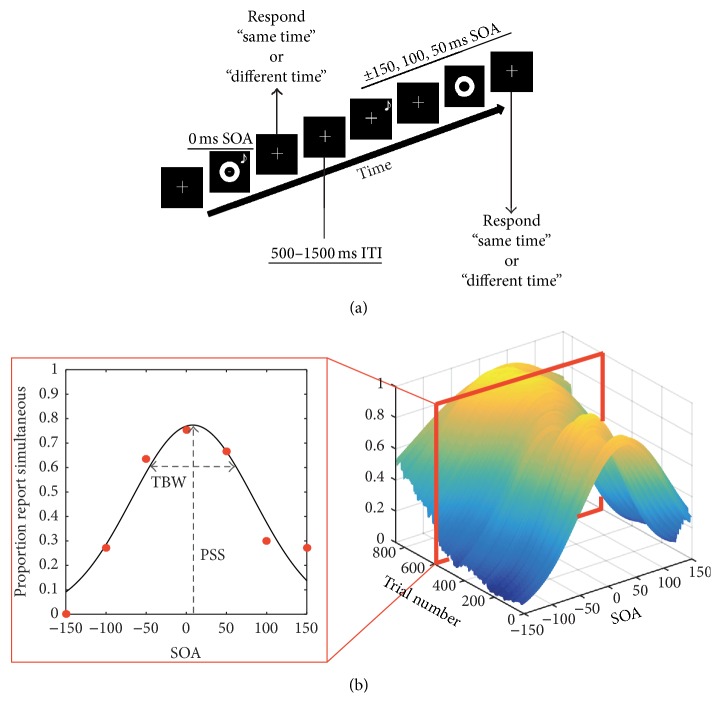
(a) Representation of a trial sequence for the simultaneity judgment (SJ) task. Participants were asked to judge if stimuli occurred at the same time or different times. (b) Individual fittings for a single participant using the sliding-window approach across Trials 1–860. The inset on the left shows a single fitting at one time-point along the time course with the PSS (mean of distribution) and TBW (standard deviation of distribution) at that particular moment in time. The TBW, PSS (cumulative) and ΔTBW, ΔPSS (rapid) were normalized on a within-subject basis, and in order to correct for multiple comparisons we consider an effect significant at *α* < 0.01 for at least 10 consecutive trials. Trial 0 was defined as the 140 trials utilized to establish initial estimates of the PSS and TBW. The time course analysis was conducted on the following 860 trials. From Trials 1–720, participants were assigned to one of four groups that received varying amounts of feedback following a response. From Trials 721–860, no feedback was presented following a response for all participants.

We employed simultaneity judgment (SJ) task to measure audiovisual temporal acuity as prior studies assessing temporal recalibration have utilized similar SJ tasks [12, 15, 22, 38, 39]. Participants were seated in a light and sound attenuating WhisperRoom™ (SE 2000 Series, Whisper Room Inc.) room for all tasks. All visual stimuli were presented at approximately 60 cm from the seated participants. A fixation marker (1 cm × 1 cm) on a black background was present on the screen both between trials and throughout the duration of a trial including presentation of the visual stimulus. Participants were asked to maintain fixation on the fixation marker throughout the experiment. For the SJ task, participants were instructed to judge whether the visual stimulus and auditory stimulus “were synchronous, at the same time” or “were asynchronous, at different times” by pressing either 1 or 2, respectively, using a keyboard (see Figure 1(a)). The visual stimulus consisted of a white ring on a black background that subtended 7.2° of visual space with an outer diameter of 6.0 cm and an inner diameter of 3.0 cm. Visual stimuli were presented for 8.3 ms (the duration of a single screen refresh cycle) on a monitor (Samsung syncmaster 22-inch 2233 RZ LCD) with a refresh-rate of 120 Hz. The auditory stimulus consisted of 1800 Hz tone that was presented biaurally via headphones (Sennheiser HD 558) with no interaural time or level differences. Auditory stimuli were 10 ms in duration (1.3 ms onset and offset ramp) and were presented at 83 dB and were calibrated using a sound level meter (Larson Davis SoundTrack® LxT2). For each trial, visual and auditory stimuli were presented in synchrony (0 ms of asynchrony between onset of visual stimuli and onset of maximal auditory amplitude) or with a stimulus onset asynchrony (SOA) ranging ±400–50 ms (negative values indicate that the auditory stimulus was the leading stimulus while positive values indicate that visual stimulus was the leading stimulus). To ensure accurate presentation of auditory and visual stimuli, SOAs were verified externally using an oscilloscope. A response screen was presented following each audiovisual pair at which time subjects could make a response. The intertrial interval (ITI) was randomly jittered from 500 to 1500 ms (uniform distribution). MATLAB (The MathWorks, Inc.) with Psychophysics Toolbox extensions [40, 41] was used to create and present the SJ task.

### 2.3. General Experimental Procedure for the Assessment of Temporal Recalibration

The estimates put forward on Trial 0 were defined as the values derived from the trials comprising the first phase of the experiment (Trials 0 to 300). The time course analysis was conducted on the following 860 trials. From Trials 1–720 of the second phase, participants were assigned to one of four groups that received varying amounts of feedback following a response (see below). From the next 140 trials, no feedback was presented following a response for all participants.

To derive initial estimates of the TBW and PSS (to assess cumulative recalibration) and trial-to-trial change in the PSS and TBW (to assess rapid recalibration), participants first completed an initial block of 300 trials (although only 140 of these were utilized, see below) of the SJ task comprising 20 trials at each of the following SOAs: ±400, 300, 250, 200, 150, 100, 50, and 0 ms. Performance over this block of trials was utilized to derive Trial 0 of the time course analysis (effects of feedback on the time course of recalibration will be tested against this Trial 0, see below). Participants subsequently completed a second block of 720 trials of the SJ task with stimuli presented at SOAs of ±150, 100, 50, and 0 ms. To avoid introducing a response bias for participants in groups receiving feedback, the number of trials at each SOA was not equally distributed in the second trial block. Instead true synchrony was overrepresented at a 6 : 1 ratio in comparison to the objectively asynchronous SOAs, such that the total number of simultaneous trials presented was equal to the total number of asynchronous trials presented (0 ms, 360 trials; ±150, 100, and 50 ms × 60 trials each). Participants next completed a third block of 300 additional trials of the SJ task identical to the first block of 300 trials (20 trials × 15 SOAs; ±400, 300, 250, 200, 150, 100, 50, and 0 ms). The time course analysis was performed using the responses in the second and third blocks. During only the second block was feedback presented for groups receiving a form of feedback. No feedback was presented during the first or third blocks. Total duration of the experiment was under 1 h30 min, and participants were given an opportunity to rest after every 100 trials in each experimental trial block.

### 2.4. Presentation of Feedback Signal

**Figure 2:**
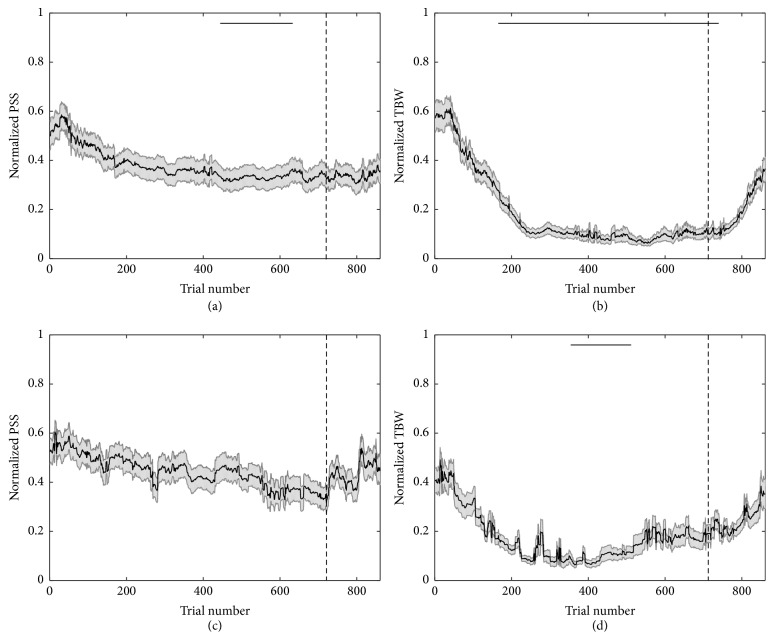
The time course of cumulative (grayscale) multisensory temporal recalibration of the PSS ((a) and (c)) and TBW ((b) and (d)) with ((a) and (b)) and without feedback ((c) and (d)). Solid bars shown above the time course are indicative of at least 10 consecutive trials at which the PSS or TBW (cumulative recalibration) significantly differed from Trial 0 (*α* < 0.01 for all trials). Shaded region illustrates SEM at each trial across the time course analysis. The post-feedback period beginning at Trial 721 is denoted by the gray, dashed vertical line.

**Figure 3:**
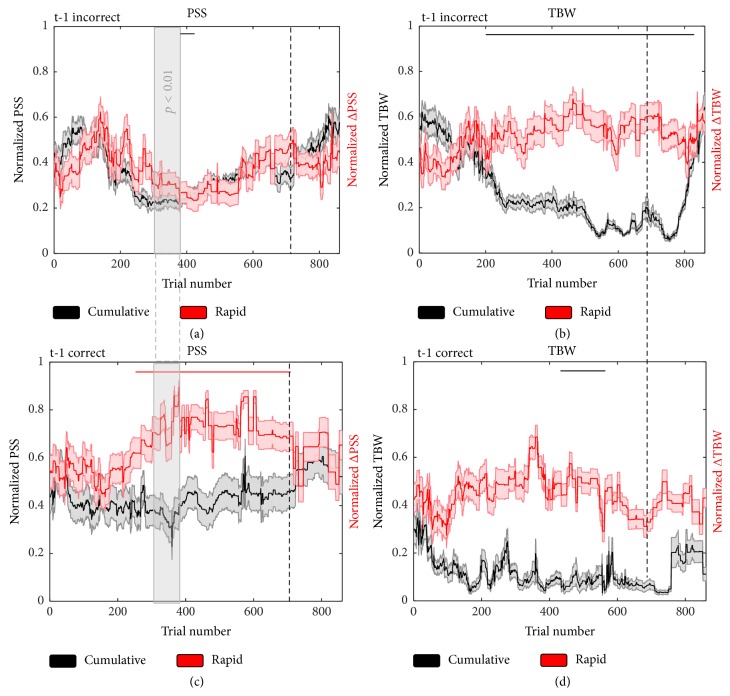
The time course of multisensory rapid (color) and cumulative (grayscale) temporal recalibration as a function of prior negative ((a) and (b)) and positive ((c) and (d)) feedback on trial t-1. Shaded region illustrates SEM at each trial across the time course analysis. Solid bars shown above the time course are indicative of at least 10 consecutive trials at which the PSS or TBW (cumulative) or ΔPSS or ΔTBW (rapid) significantly differed from Trial 0 (*α* < 0.01 for all trials). The trials for which we observed a significant interaction of temporal recalibration (cumulative versus rapid) × feedback (t-1 correct versus t-1 incorrect) is indicated by the vertical solid gray shading (*α* < 0.01 for all trials).

**Figure 4:**
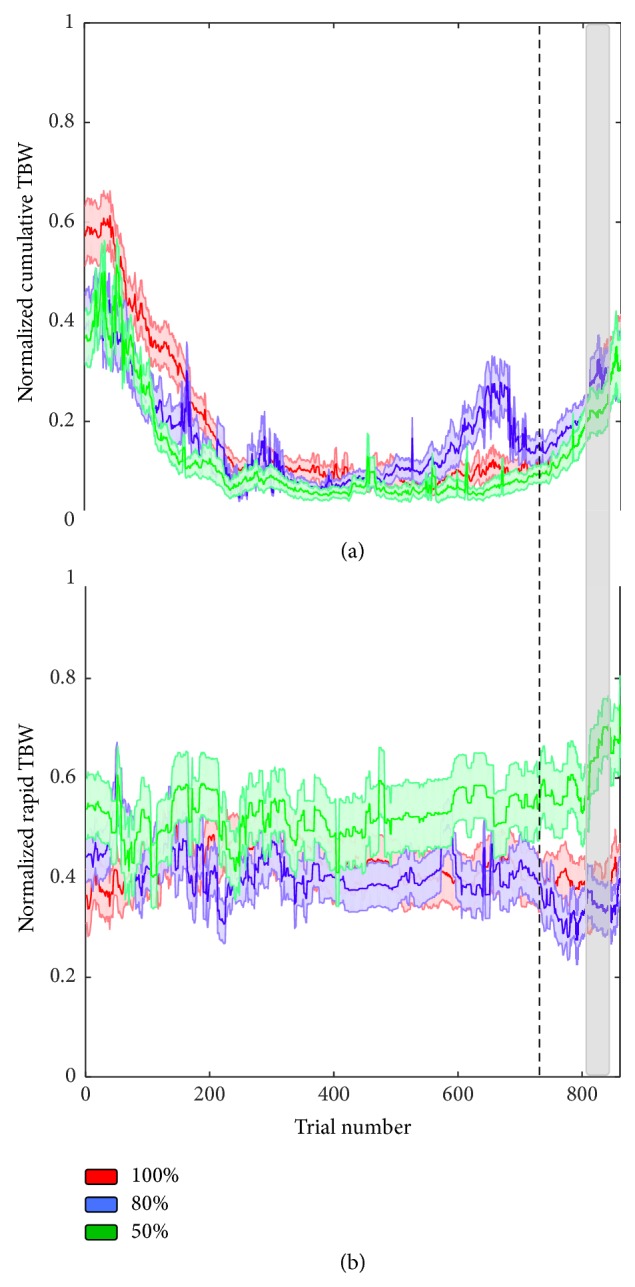
The time course of cumulative (a) and rapid (b) temporal recalibration of the TBW for feedback that was 100% (red), 80% (blue), and 50% (green) reliable. Shaded region illustrates SEM at each trial across the time course analysis. We observe that the time course of rapid recalibration of the TBW for the group receiving uncorrelated feedback signals (50% reliable) diverges from the other group in the post-feedback trial block such that there is greater trial-to-trial readjustment of the TBW (the area of vertical gray shading indicates interaction).

For all participants, feedback was not provided during the presentation of trials in the first trial block. Participants were randomly assigned to one of four experimental groups characterized by the nature of the feedback presented during the second block. For the first group (*n* = 15), participants did not have access to any explicit feedback. Participants in the second group (*n* = 25) had access to reliable visual feedback in the form of a blue-green check mark or red X following objectively correct and incorrect responses. Reliable feedback was defined as feedback that accurately reflected the objective relationship of the audiovisual stimuli. The third (*n* = 13) and fourth (*n* = 12) groups were, respectively, presented with reliable feedback on only 80% and 50% of trials (i.e., false or erroneous feedback on 20% and 50% of the remaining trials). False feedback (i.e., feedback that was not reliable) was defined as the presentation of the incorrect feedback for each SOA-response pair (i.e., a response of synchronous for a trial in which the SOA presented was 0 ms yielded the presentation of a red X, the exact opposite of the objectively accurate feedback). Reliable and false feedback were distributed equally between synchronous and asynchronous trials for all participants. All feedback was presented for 500 ms immediately following the participant's response. No feedback was presented for the third trial block for all participants (post-feedback period as denoted by dashed line at Trial 721 in Figures 2, 3, and 4 and Figures S1 and S2 in Supplementary Material available online at https://doi.org/10.1155/2017/3478742).

### 2.5. Analysis of the Time Course of Multisensory Temporal Recalibration

Two distinct multisensory temporal recalibration time courses are of interest here. The first, denominated “cumulative” recalibration, refers to the degree to which participants consider accumulating feedback when executing audiovisual simultaneity judgments. This recalibration, thus, requires the conscious acknowledgment of received feedback. The second, referred to as “rapid” recalibration, denotes the degree to which the nature of the immediately preceding trial (t-1)—audio- or visual-leading—influences the perception of simultaneity at the given trial (t). The examination of rapid audiovisual temporal recalibration effects, thus, is taken to index an implicit sensory phenomenon, perceptual learning, and involves a one-back analysis (analysis of trial t as a conditional of trial t-1).

PSS and TBW for the different conditions and time courses were contrasted. In order to examine the effect of feedback, we compared the mean initial estimate of PSS, ΔPSS (i.e., rapid change in PSS), TBW, and ΔTBW (i.e., the estimates based on block 1: no feedback) to estimates derived from subsequent time period with feedback (a sliding time window of 140 trials, see below). To maintain a consistent estimate of the different parameters exposed above across all blocks, only SOAs of ±150, 100, 50, and 0 ms were utilized to fit distributions of reports of synchrony as a function of SOA for the entire time course. Although we employed wider ranging SOAs in the first trial block to ensure an accurate estimate of the PSS and TBW, further analysis revealed that fitting these distributions with the entire course of SOAs and those present across all trial blocks did not result in any significant differences in our initial estimates for all measures (*p* > 0.21 for all measures). Thus, initial distributions of reports of synchrony as a function of SOA (Trial 0 in the time course analysis) were drawn based on the 140 trials comprising the first trial block (no feedback). These distributions of responses were fitted with a Gaussian distribution whose amplitude, mean, and standard deviation were free to vary (see (1)).

(1) $$\begin{array}{c} P\left(\;response\;|\;SOA\;\right)=\text{amp}\times {exp}^{-\left({\left(SOA-PSS\right)}^{2}/{2SD}^{2}\right)}. \end{array}$$

The normal distribution proved to be overall a good fit (mean *R*^2^ = 85.6, SD = 2.45). The mean of the best fitting distribution is taken as the PSS and the standard deviation as a measure of the TBW. That is, PSS is the point (i.e., SOA) at which participants are most likely to categorize a presentation as synchronous and the TBW is the temporal interval over which participants are highly likely to categorize the presentation as synchronous.

In order to index rapid recalibration, the amount of change in these values (PSS and TBW) is computed (e.g., ΔPSS = PSS audio-leading − PSS visual leading) as a function of the prior trial. Further, in order to examine the time courses of rapid versus slow multisensory temporal recalibration effects, we adopt a sliding-window approach (Figure 1(b)). That is, after the first estimation of the mean and standard deviation of the Gaussian describing reports of synchrony, a window of 140 trials—initially placed between Trials 0 and 140—is moved trial-per-trial across the entire span of the second trial block (Trials 1–720) as well as during the third trial block post-feedback (Trials 721–860). A window of 140 trials is chosen in order to mimic the initial estimates based on the first block of 140 trials. At each subsequent step the new distribution is fitted again, and estimates of the mean and standard deviation are calculated. Similarly, at each step rapid recalibration values are recomputed. Upon completion of the protocol (~5000 fittings per subject), PSS and TBW values were normalized from 0 to 1 within-subjects. That is, in order to appropriately compare across time scales of recalibration (cumulative versus rapid), with vastly different values and ranges, the data were normalized. For every participant and for each of their parameters (PSS, ΔPSS, TBW, and ΔTBW), their time-series was normalized such that their most extreme absolute values, minimum and maximum, respectively, corresponded to a value of 0 and 1, respectively. Absolute values were taken in order to assure interpretability of the PSS estimates. That is, for the TBW, there is no possibility of negative values; thus the smaller the window is, the closer our normalized estimate is to 0, whether absolute values are taken or not. For the PSS, however, negative values are possible. Here, however, we are interested in determining the relationship of a particular individual's PSS to true synchrony, SOA = 0 ms. Hence, we take the absolute value. Deriving these normalized values allows for comparison across time scales and participants but undoubtedly obfuscates interpretation. To be clear, this data is normalized within subjects, and hence, when PSS approaches 0, it does not mean that the group's PSS was equal to zero, but that at this instance all or most participants were at their smallest PSS (in absolute value). Similarly, a TBW of or close to zero indicates the smallest TBW reached for each individual—a normalized TBW of 0.1 is more precise than a TBW of 0.2 but delineates no absolute measure of “preciseness.”

The effects of feedback on recalibration (i.e., analyses comprised in Figure 2; first section) are analyzed via a one-sample *t*-test versus the initial estimate of the given parameter (i.e., estimate on Trial 0). The impact of the nature of the feedback (positive versus negative; second section) on recalibration is analyzed via one-sample *t*-test (as above) and via within-subjects ANOVAs where appropriate (see below). Similarly, for the analyses regarding the reliability of feedback (i.e., third section), results are analyzed with both one-sample *t*-test and within- and between-subjects ANOVAs, where appropriate. Given the inherent multiple comparisons problem in utilizing a sliding-window approach, we correct for false positives by considering an effect significant by setting *α* < 0.01 for at least 10 consecutive window positions (see [25] for a similar approach).

## 3. Results and Discussion

### 3.1. Feedback Accelerates and Maintains Cumulative Temporal Recalibration

Previous adaptation studies have shown that temporal recalibration occurs over slow time scales as extended periods of passive exposure to asynchronous stimuli (often biased in the direction of either an auditory or visual leading stimulus with a constant SOA) elicit changes in perceptual representations as indexed via the PSS and TBW (audiovisual, see [12, 13, 15, 22–24, 28, 42, 43]; for other modalities see [15]). Here, we sought to address if sensory experience for unbiased, asynchronous stimuli elicited changes in time course of the PSS or TBW based on immediately prior (rapid calibration) or cumulative (cumulative recalibration) sensory history. We first sought to assess the cumulative time course of temporal recalibration in the presence or absence of feedback as participants completed the SJ task (i.e., feedback, in this initial comparison, was reliable on 100% of trials). In the presence of feedback, the PSS, as illustrated in Figure 2(a), decreased in absolute value over time. This shift in the PSS toward objective synchrony (i.e., an SOA of 0 ms) became significant for the interval between Trials 413 and 623 (black bar denotes period of significant difference, one-sample *t* test; *t*(24) => 2.49, *p* < 0.01, partial eta-squared => 0.09). In contrast, the absence of feedback (Figure 2(c)) failed to result in any significant changes in the PSS over the course of the experiment (all *p* ≥ 0.04). These results illustrate that feedback coupled to the presentation of sensory information, but not sensory statistics alone, was responsible for the shift in the PSS toward objective synchrony.

With regard to the TBW, as illustrated in Figures 2(b) and 2(d), there was significant narrowing for groups receiving feedback ((a) and (b), significant at *p* < 0.01 between Trials 170 and 720, one-sample *t* test, *t*(24) => 2.49, partial eta-squared => 0.09) as well as for those who did not ((c) and (d), significant between Trials 370 and 540, *t*(24) => 2.49, partial eta-squared => 0.09). These results highlight a cumulative recalibration of audiovisual temporal acuity, even under circumstances of more passive sensory stimulation. However, the dynamics of these changes differed between the feedback and no-feedback conditions, with the narrowing arising more rapidly in the presence of feedback and persisting until the end of the feedback epoch (as opposed to the transient effect observed in the case of no feedback).

In addition to examining the effects during the training interval (i.e., the period in which feedback was given, Trials 1–720), we also sought to examine the durability of these effects following the removal of feedback. This assessment was carried out over Trials 721–860. For individuals previously given feedback, the earlier effect of cumulative recalibration of the TBW persisted over Trials 721–744 (*t*(24) => 2.49, *p* < 0.01) but dissipated with time. For the group that did not receive feedback, no change in the time course of cumulative recalibration was observed during this period (Trials 721–860, all *p* ≥ 0.29). For the PSS, not surprisingly we did not observe any additional changes in the time course of cumulative recalibration during this period for both the group receiving feedback and the group that did not receive feedback (all *p* ≥ 0.20).

In contrast to these cumulative recalibration effects on both the PSS and TBW, little was seen in regard to a change in the time course of rapid recalibration with regard to the initial estimate of rapid recalibration at Trial 0 (see Figure S1). Thus, when the data were analyzed on the basis of the immediately preceding trial (audio- versus video-leading), there were no apparent changes in the time course of recalibration effects for either the PSS or the TBW, or for the feedback and no-feedback groups (all *p* ≥ 0.34). We did, however, observe that, for the mean of all trials, the PSS (Feedback* M *(mean) = 14.8 ms, one-sample *t*-test to zero; *t*(24) =< 3.46, *p* < 0.001, partial eta-squared => 0.17; No Feedback* M* = 11.1 ms, *t*(14) =< 3.78, *p* < 0.001, partial eta-squared => 0.35) and TBW (Feedback* M* = 5.1 ms, *t*(24) =< 3.46, partial eta-squared => 0.17, *p* < 0.001; No Feedback* M* = 5.1 ms, *t*(14) =< 3.78, partial eta-squared => 0.35, *p* < 0.001) were significantly shifted on a trial-to-trial basis. Thus, while a significant effect of rapid recalibration is present between individual trials, the magnitude of the recalibration does not change when analyzed as a time course. Hence, we conclude that while immediately prior sensory experience (i.e., bottom-up factors) shifted the PSS and TBW on a trial-to-trial basis, sensory experience alone is not sufficient to influence a change in the time course of rapid recalibration.

Collectively, the results illustrate that change in sensory statistics alone is enough to drive perceptual learning, as defined by the cumulative narrowing of the TBW in the no-feedback group. However, the time course of this plasticity is accelerated when feedback was provided. Further, changes in the PSS, which took place over a slower time scale and were more transient when compared to the TBW, indicated that this measure is more stable as compared to the TBW (a finding reinforced by the lack of change for the PSS in the no-feedback conditions). Although prior work has shown perceptual learning in the absence of a reinforcement signal [12, 13], the enhanced temporal recalibration observed when a feedback signal is present resembles the enhancing effect of feedback for other forms of perceptual learning [16, 17]. Thus, while perceptual learning may occur over time, feedback accelerates perceptual learning. The capacity for feedback to elicit more rapid temporal recalibration in response to feedback is likely adaptive as it would allow for faster changes in perception that would allow for more accurate responses to the salient aspects of the sensory environment. For other indices of temporal perception, as we observe for the PSS, feedback may be essential for perceptual learning to occur [33, 44], although our data does not preclude temporal recalibration of the PSS with increasing sensory exposure.

### 3.2. Positive and Negative Feedback Differentially Impact the Time Course of Temporal Recalibration

As illustrated above, feedback strongly influences the time course of cumulative audiovisual temporal recalibration. However, how this feedback is driving these changes remains an open question. Stated differently, individuals received two forms of feedback in the context of this task—positive feedback when they were correct in their judgment and negative feedback when they were incorrect in their judgment. Do these two types of feedback differentially impact the time course of temporal recalibration? That such a distinction might exist is grounded in evidence from studies of reward system circuitry, which show that this system is differentially activated by positive and negative feedback and is underpinned by distinct neural networks [10]. Additionally, although no change was seen in rapid recalibration in the presence of feedback, this initial analysis lumped together positive and negative feedback, which may have masked differential effects based on prior feedback history. Hence, we analyzed both cumulative and rapid recalibration effects of the PSS and TBW as a function of whether individuals were correct (i.e., received positive feedback) or incorrect (i.e., received negative feedback) on the previous trial. That is, in order to assess if the time course of temporal recalibration was affected by positive or negative feedback, distributions of perceived simultaneity (i.e., report of synchrony) as a function of SOA were compiled for each participant separately for the cases in which on the precedent trial (t-1) participants were informed that their answer had been correct (t-1 correct; prior positive feedback) or incorrect (t-1 incorrect; prior negative feedback). Additionally, in order to compute rapid recalibration effects, reports of synchrony were further bifurcated into those in which trial t-1 had either a negative (i.e., audition led) or positive SOA (i.e., vision led).

Findings revealed a relatively small effect of feedback type on the dynamics of PSS cumulative recalibration. Negative feedback drove a very transient change in the PSS toward true synchrony (i.e., smaller absolute value; significant at *p* < 0.01, *t*(24) => 2.49, partial eta-squared => 0.11, between Trials 392–409; Figure 3(a)). Positive feedback did not elicit significant cumulative recalibration of the PSS (Figure 3(c), black lines). In contrast, for the TBW, cumulative recalibration was greatly impacted by feedback. Following prior incorrect responses (i.e., negative feedback), narrowing of the cumulative TBW was evident earlier and sustained over a longer time course (Trials 200–720, *p* < 0.01, *t*(24) => 2.49, partial eta-squared => 0.11) than changes to the TBW observed following prior correct responses (i.e., positive feedback) (Trials 418–551; Figures 3(b) and 3(d)). Collectively, these results support the conclusion that feedback that informs (i.e., incorrect feedback), rather than confirms (i.e., correct feedback), a perceptual decision accelerates and sustains perceptual learning.

For rapid recalibration, immediately preceding positive feedback elicited a significant change in PSS that began relatively early and lasted for the duration of the feedback (i.e., significant, *p* < 0.01, *t*(24) => 2.49, partial eta-squared => 0.11, change in PSS between Trials 239 and 720 for t-1 correct trials; Figure 3(c), red lines). Thus, it appears that, following a signal confirming a perceptual decision, individuals exhibited a greater propensity for adjusting their PSS on a trial-by-trial basis. In contrast, no significant change in the PSS was seen after negative feedback (all *p* ≥ 0.39). No change in rapid recalibration of the TBW was observed as a function of positive or negative feedback (all *p* > 0.52).

In order to examine the interaction between cumulative and rapid recalibration effects as a function of feedback type, separate 2 (cumulative versus rapid) × 2 (previous trial correct versus incorrect) within-subjects ANOVAs for the PSS and TBW were conducted. As illustrated in Figure 3, a significant interaction was observed for the PSS (Trials 301–399, *p* < 0.01, *F*(1, 96) => 6.91, partial eta-squared => 0.06, as illustrated by the gray shaded area). This effect was driven by the finding that when on the previous trial participants had been informed of an incorrect response, the time courses of cumulative and rapid recalibration followed one another. This was not the case when the participant had been informed of a correct response on the previous trial. Hence, when participants were informed of a correct response on the preceding trial, they appear to more readily incorporate recent sensory evidence into their judgments. Summarizing these results, under conditions of informative (i.e., negative) feedback, the time courses of rapid and cumulative recalibration appear to be yoked, while, under conditions of confirmative (i.e., positive) feedback, rapid and cumulative recalibration effects appear to uncouple. This uncoupling may be adaptive in that only corrective signals are able to drive rapid plasticity.

### 3.3. Time Course of Rapid Recalibration of the TBW Diverges as a Function of Prior Feedback Reliability

To better understand the contribution of feedback to recalibration processes and the interrelationship between rapid and cumulative recalibration effects, we tested whether changing the reliability of the feedback would differentially alter rapid versus cumulative temporal recalibration. Prior studies of visual perceptual learning have demonstrated that while feedback enhances perceptual learning, presenting feedback that is uncorrelated to responses (i.e., unreliable feedback) impairs perceptual learning [45]. Feedback was provided to different groups of participants and was reliable on 100%, 80%, or 50% of trials (for this comparison, the group receiving 100% reliable feedback was the same group of participants that was previously compared to the no-feedback condition; see Section 3.1). We hypothesized that if the time course of temporal recalibration was dependent on external reinforcement, we would see progressively less temporal recalibration as feedback reliability decreased. Indeed, unlike the group receiving 100% reliable feedback (described above), we did not observe cumulative recalibration of the PSS for the groups receiving 80% or 50% reliable feedback (see Figure S2). In contrast, however, we did observe cumulative recalibration of the TBW for all groups, although these changes were seen over a shorter extent of trials when compared with the 100% reliable feedback group (see Figure S2). Specifically, when participants were 100% reliably informed of their performance, TBWs were significantly smaller during and after feedback than before feedback between Trials 170 and 744 (all *p* < 0.01). In the cases of 80% and 50% reliable feedback, the feedback effects were somewhat more short-lived (resp., between Trials 305 and 541, *p* < 0.01, and between Trials 219 and 430, *p* < 0.01), nonetheless apparent. These findings are similar to the transient change in the TBW without any changes in the PSS that was observed in the absence of feedback (see Section 3.1).

In order to examine the different time courses of multisensory temporal recalibration as a function of feedback reliability, we conducted separate 2 (type of recalibration: cumulative versus rapid) × 3 (feedback reliability: 100%, 80%, 50%) between-subjects ANOVAs for the PSS and TBW along the time-series of trials such that an effect was interpreted as significant at *α* < 0.01 for at least 10 consecutive trials. For the PSS, we did not observe a main effect of type of recalibration, feedback reliability, or an interaction (see Figure  S2, all *p* ≥ 0.24). When this analysis was expanded to include the no-feedback group, conducting a 2 (type of recalibration: cumulative versus rapid) × 4 (feedback reliability: 100%, 80%, 50%, no feedback) mixed model ANOVA on PSS values did not alter the above-mentioned findings (all *p* > 0.18). In contrast, for the TBW, we observed a significant main effect of type of recalibration between Trials 103 and 841 (all *p* < 0.01, *F*(1, 294) => 6.72, partial eta-squared => 0.04) and a significant type of recalibration × feedback reliability interaction between Trials 816 and 844 (Figure 4; *F*(2, 294) => 4.68, all *p* < 0.01, partial eta-squared => 0.03; indicated by the gray shading). Thus, and as is evident in Figure 4, although the dynamics of cumulative temporal recalibration of the TBW failed to differ dependent upon feedback reliability ((a); one-way between-subjects ANOVA, all *p* > 0.06), the dynamics of rapid temporal recalibration of the TBW did diverge (b). Specifically, a one-way between-subject ANOVA on the rapid recalibration values demonstrated a significant effect between Trials 806 and 851 (*F*(2, 47) => 5.09, all *p* < 0.01, partial eta-squared => 0.17). Subsequent post hoc *t*-tests performed on the rapid recalibration patterns as a function of feedback reliability demonstrated that the 50% reliable feedback elicited a higher degree of rapid recalibration (variability on a trial-by-trial basis, weighting more heavily immediately preceding sensory experience) than the 80% reliable feedback (between Trials 780 and 861, *t*(23) => 2.50, *p* < 0.01, partial eta-squared => 0.21) and the 100% reliable feedback (between Trials 801 and 827, *t*(35) => 2.43, *p* < 0.01, partial eta-squared => 0.14). The 100% and 80% reliable feedback conditions did not differ from one other (all *p* > 0.33). Additionally, when performing one-sample *t*-test to their respective departing values after the no-feedback phase (e.g., Trial 0) the 50% reliable group demonstrated a significant increase in TBW rapid recalibration (Trial 556 onward, *t*(11) => 2.71, *p* < 0.01, partial eta-squared => 0.4), while the 80% and 100% reliable groups showed no change (all *p* > 0.03).

Enlarging this analysis in order to include the no-feedback group and conducting a 2 (type of recalibration: cumulative versus rapid) × 4 (feedback reliability: 100%, 80%, 50%, no feedback) mixed model ANOVA on TBW values conserved the presence of a main effect of type of recalibration between Trials 117 and 841 (all *p* < 0.01, *F*(3, 294) ≥ 5.12, partial eta-squared ≥ 0.06) and a significant type of recalibration × feedback reliability interaction between Trials 816 and 844 (*F*(4, 294) ≥ 3.18, all *p* < 0.01). Feedback reliability groups did not differ from one another with regard to the time course of cumulative recalibration (between-subjects one-way ANOVA; all *p* > 0.03) but did regarding the time course of rapid recalibration (between-subjects one-way ANOVA, *p* < 0.01) between Trials 806 and 851 (as mentioned above). Subsequent post hoc *t*-test showed that the no-feedback group differed from the 100% (*p* < 0.01, more trial-to-trial recalibration in the no-feedback group between Trials 825 and 860), the 80% (*p* < 0.01, more trial-to-trial recalibration in the no-feedback group between Trials 818 and 860), and the 50% reliability groups (*p* < 0.01, less trial-to-trial recalibration in the no-feedback group between Trials 809 and 827).

The increase in rapid recalibration when feedback is not present during the post-feedback trial block for the group that had previously been presented with the 50% reliable feedback signal may represent an increased tendency for the subjects to disregard feedback and more heavily weigh sensory statistics when prior feedback has been unreliable in signaling the correctness of their judgments.

This finding represents the second example in our data of an uncoupling between cumulative and rapid recalibration (the first being that brought about by the correct versus incorrect nature of the feedback). Namely, we observe that when feedback reliability is reduced, perceptual learning occurs, but with differing dynamics for cumulative and rapid recalibration, again suggesting differing mechanistic processes. We hypothesize that, as a result of the conflict between sensory evidence and feedback signals, those individuals presented with the least reliable feedback (50%) were more likely to rely on immediate sensory information to recalibrate their audiovisual temporal representation. This may be due to a decreased reliance on top-down signals generated by sensory feedback and an increased reliance on bottom-up sensory information. In the groups receiving unreliable feedback, as some of the feedback was misinformative, increased reliance on sensory statistics would be adaptive in that sensory driven recalibration would produce a more accurate perceptual representation.

## 4. General Discussion

Here we show that top-down factors (i.e., feedback signals) can interact with bottom-up signals in order to change the dynamic time course of temporal recalibration for two measures of audiovisual temporal perception (PSS and TBW). By employing a sliding-window analysis for this study, we were able to characterize, for the first time, how rapid and cumulative temporal recalibration occur in both the presence and absence of feedback and to characterize the differing temporal dynamics for these two time scales of perceptual learning. Our findings illustrate that while sensory experience alone is sufficient to elicit some degree of temporal recalibration, feedback signals can work in conjunction with sensory experience to produce greater perceptual plasticity.

That feedback signals alter the dynamics of temporal recalibration is not surprising as enhanced plasticity would be adaptive in response to changing environmental statistics or task demands. Despite this assumption, it is interesting that feedback is sufficient, if only transiently, to alter perceptual representations for which a strong history of sensory experience exists. The PSS, a measure that is reflective of an individual's internal representation of the temporal statistical structure of the external world, is rarely at true synchrony (i.e., 0 ms). Rather, this measure is typically biased toward an asynchrony in which the visual stimulus leads the auditory stimulus—reflective of the typical statistical structure of audiovisual stimuli within our world [30, 32, 34, 46]. Although adaptation studies have shown that repeated presentation of asynchronous audiovisual stimuli (i.e., toward either a visual or auditory leading stimulus set) can shift the PSS in the direction of the experienced asynchrony [47–49], we report a shift in the PSS in the absence of any changes in the temporal structure of the stimuli and based solely on the presence of feedback. Indeed, the changes elicited under such circumstances are invariably in the direction of true synchrony. As we did not introduce a change in the temporal structure of the stimuli that would favor a directional shift in the PSS, we conclude that this change is driven largely by top-down factors linked to the delivery of feedback.

That the changes in TBW and PSS in response to feedback are quick to develop is also not surprising as this too may be adaptive. Interestingly, it also appears that, over the course of a single session, both sensory and feedback-induced changes in the PSS and TBW can be quick to dissipate as, with the exception of the group receiving 100% reliable feedback, the time course returns to the level of the initial estimate within a relatively small number of trials after feedback is removed. As studies of perceptual training have reported changes in temporal acuity between training sessions [50, 51], it is possible that by extending our analysis across multiple sessions we might observe further changes in the time course of recalibration. Future investigations will be necessary to determine if sensory experience or feedback elicits durable changes in the PSS and TBW or whether the plasticity we observe is simply reflective of fast adaptation.

Future studies may also explore if unreliable feedback elicits lasting changes beyond the post-feedback period measured in this study and if such changes differ from any changes elicited by sensory experience in the absence of feedback. Although we observed similarities in the time course of recalibration of the TBW and PSS in the absence of feedback and with unreliable feedback, the mechanism by which these changes occur may be different. It is possible that the return to original levels in performance we observed may be due to a change in criteria when unreliable feedback was present (i.e., Trials 1–720) rather than perceptual learning that results in a lasting change in the perceptual representation [52]. Changing perceptual decision criteria in response to erroneous (i.e., unreliable) feedback has been suggested to be adaptive as such a transient change in criteria would minimize error signals while protecting prior representations of the stimuli [53]. Thus, when the unreliable feedback signal is removed, the criterion can be rapidly adjusted to criterion prior to exposure to unreliable feedback. Although changes in criterion are usually limited to a perceptual training session, changes in criterion are typically not observed during a second session a day later whereas changes in sensitivity are maintained after at least a day [54]. By extending the time course analysis of temporal recalibration beyond a single day, we would hypothesize that if unreliable feedback elicited any durable change in the PSS or TBW, we would observe a change in the PSS or TBW relative to the final estimate of the TBW or PSS on the first day.

Increasing evidence suggests that the mechanisms supporting unisensory (i.e., within-modality) perceptual learning are evident at higher cortical levels [33, 55] and that enhanced perception of amodal sensory properties due to perceptual training in modality can exhibit transfer across sensory modalities to an untrained sensory modality [56, 57]. Stimulus exposure that is more passive in nature appears to drive changes at lower cortical levels while increasingly the relevance of the stimulus properties elicits changes at both higher and lower cortical levels [8]. Multisensory stimuli, which engage a larger cortical network than unisensory stimuli, may facilitate perceptual learning by increasing activity of primary sensory regions as well as higher-level sensory cortex. Recent evidence suggests that multisensory interactions, while present across different levels of the cortical hierarchy, may differ in their computational functions across higher-order and sensory regions [12, 13]. Accordingly, a feedback signal may also serve to engage a larger cortical network, which in turn enables a greater capacity for perceptual learning to occur.

A possible explanation for why greater temporal recalibration occurs with feedback, provided it is reliable, is that sensory readout is improved for higher-order cortical areas involved in sensory decision-making due to the feedback signal. At the neural level, this is in line with studies of visual perceptual learning that observed changes in activity patterns in the anterior cingulate cortex to track changes in decision-making during visual perceptual learning [34, 37]. Furthermore, neural evidence suggests that prediction error signals during perceptual learning refine and strengthen neural connectivity between sensory neurons and those neurons required for the perceptual response and thus may support changes in higher-order regions [58]. Thus, in the absence of an informative reinforcement signal, rapid but transient changes in perceptual plasticity are likely due to changes in low-level sensory areas. Future investigations will be necessary to determine if changes in the connectivity of higher-order cortical areas and low-level sensory processes underlie the observed changes in temporal recalibration and if these changes are durable or transient (see [58] for a helpful review in this regard).

## 5. Conclusions

We report that sensory experience and feedback signal interact to drive both rapid and cumulative temporal recalibration of the TBW and PSS for audiovisual stimuli. While rapid and cumulative temporal recalibration often follow similar time courses, these time courses may diverge dependent upon prior feedback signals. Our findings support the fact that prior sensory history feedback signals influence subsequent perceptual plasticity to elicit both rapid and cumulative temporal recalibration.

## Supplementary Material

Provided in the Supplementary Material are the time course of rapid recalibration with and without feedback (S1) as well as the individual time course for rapid and cumulative recalibration for the 100%, 80%, and 50% reliable feedback groups (S2).

## Data Access

Data from this study can be viewed at https://vanderbilt.box.com/s/a04pr5dbsjrhrnquz5lrcvv5i6buafdp.
