# Supplementary material for: The Impact of Feedback on the Different Time Courses of Multisensory Temporal Recalibration
Source: Neural Plast. 2017 Feb 21;2017:3478742. doi: 10.1155/2017/3478742 (PMC5339631; doi:10.1155/2017/3478742)
Supplement: Supplementary file 1 — Provided in the Supplementary Material are the time course of rapid recalibration with and without feedback (S1) as well as the individual time course for rapid and cumulative recalibration for the 100%, 80%, and 50% reliable feedback groups (S2). [file 3478742.f1.docx]

**Figure S1**. The timecourse of rapid temporal recalibration in the presence and absence of feedback. The normalized magnitude of trial-to-trial change (i.e. rapid recalibration) in the PSS (left) and TBW (right) did not change over the course of the second trial block (Trials 1-720) when feedback was either present (red) or absent (yellow). Similarly, the magnitude of rapid recalibration remained stable when feedback was removed for the one group during the third trial block (Trials 721-860; right of dashed line). While we did not observe a change in the magnitude of rapid recalibration, the PSS (Feedback *M* = 14.8, *p* < .001; No Feedback *M* = 11.1, *p* < .001) and TBW (Feedback *M* = 5.1, *p* < .001; No Feedback *M* = 5.1, *p* < .001) were significantly shifted trial-to-trial consistent with prior reports [1, 2] of the overall rapid temporal recalibration observed over the course many trials. This analysis corresponds to the time course of cumulative temporal recalibration in Figure 2.

**Figure S2.** The time course of cumulative (gray scale) and rapid recalibration (color) for participants receiving 100% (red), 80% (blue), and 50% (green) feedback during the second trial block (Trials 1-720) and when feedback is removed (Trials 721-860). We did not observe cumulative recalibration of the PSS (left) for either group receiving unreliable (80% or 50% reliable; middle and bottom row) feedback. This lack of change in cumulative recalibration for the PSS is comparable to the effects seen in individuals without access to feedback, but differs strikingly from those in which the feedback was 100% reliable (top row). With regard the TBW (right), both groups receiving 80% and 50% reliable feedback exhibited transient cumulative temporal recalibration resulting in significant narrowing of the TBW (Trials 305-541 and Trials 219-430 respectively) which was unlike the timecourse exhibited by those for which feedback was reliable on 100% of trials (trials 170 to 720). Thus, the timecourse of temporal recalibration when feedback is less reliable resembles the timecourse observed when feedback is absent. A significant change in the magnitude of rapid recalibration was observed only for the group receiving 50% reliable feedback during the second trial block (green) after the unreliable feedback signal was removed (Trials 808-860). No change in the magnitude of rapid recalibration was observed for the groups receiving either 100% or 80% reliable feedback. Solid bars shown above the timecourse are indicative of at least 10 consecutive trials at which the PSS or TBW (cumulative) or ΔPSS or ΔTBW (rapid) significantly differed from Trial 0 (α< 0.01 for all trials).

**Supplemental References**

1. Van der Burg, E., Alais, D., and Cass, J. (2013). Rapid Recalibration to Audiovisual Asynchrony. Journal of Neuroscience *33*, 14633-14637.

2. Van der Burg, E., Orchard-Mills, E., and Alais, D. (2015). Rapid temporal recalibration is unique to audiovisual stimuli. Experimental brain research *233*, 53-59.
